# Genome-wide association mapping of flowering time and northern corn leaf blight (*Setosphaeria turcica*) resistance in a vast commercial maize germplasm set

**DOI:** 10.1186/1471-2229-12-56

**Published:** 2012-04-30

**Authors:** Delphine Van Inghelandt, Albrecht E Melchinger, Jean-Pierre Martinant, Benjamin Stich

**Affiliations:** 1 Institute of Plant Breeding, Seed Science, and Population Genetics, University of Hohenheim, Germany; 2 Limagrain Europe, Bâtiment 1, CS 3911, France; 3 Current address: Limagrain GmbH, Breeding Station, Schönburg 6, Germany; 4 Max Planck Institute for Plant Breeding Research, Carl-von-Linne-Weg 10, Germany

## Abstract

**Background:**

*Setosphaeria turcica* is a fungal pathogen that causes northern corn leaf blight (NCLB) which is a serious foliar disease in maize. In order to unravel the genetic architecture of the resistance against this disease, a vast association mapping panel comprising 1487 European maize inbred lines was used to (i) identify chromosomal regions affecting flowering time (FT) and northern corn leaf blight (NCLB) resistance, (ii) examine the epistatic interactions of the identified chromosomal regions with the genetic background on an individual molecular marker basis, and (iii) dissect the correlation between NCLB resistance and FT.

**Results:**

The single marker analyses performed for 8 244 single nucleotide polymorphism (SNP) markers revealed seven, four, and four SNP markers significantly (*α*=0.05, amplicon wise Bonferroni correction) associated with FT, NCLB, and NCLB resistance corrected for FT, respectively. These markers explained individually between 0.36 and 14.29% of the genetic variance of the corresponding trait.

**Conclusions:**

The very well interpretable pattern of SNP associations observed for FT suggested that data from applied plant breeding programs can be used to dissect polygenic traits. This in turn indicates that the associations identified for NCLB resistance might be successfully used in marker-assisted selection programs. Furthermore, the associated genes are also of interest for further research concerning the mechanism of resistance to NCLB and plant diseases in general, because some of the associated genes have not been mentioned in this context so far.

## Background

*Setosphaeria turcica* (anamorph *Exserohilum turcicum*, formerly known as *Helminthosporium turcicum*) is a fungal pathogen that causes northern corn leaf blight (NCLB) in maize. NCLB is a serious, omnipresent foliar disease [1,2]. Infections of maize with NCLB before silking can cause grain yield losses of more than 50%, which are accompanied by a reduction in feed value and the predisposition of infected plants to stalk rot [3].

Plants have evolved qualitative and quantitative resistance to combat pathogens. Qualitative resistance typically confers a high level of resistance, is usually race specific, and is based on single, mostly dominantly acting genes (R genes; for review see [4]). For NCLB, qualitative resistances have been identified and called *Ht* genes (for *Helminthosporium turcicum*): *Ht1*[5] and *HtP*[6] were mapped to the long arm of chromosome 2, *Ht2*[7] as well as *Htn1*[8] were mapped to the long arm of chromosome 8, and *Ht3* was the only resistance gene that was ever introgressed from *Tripsacum floridanum* into maize [9]. These single resistance genes have been backcrossed into a number of widely used inbred lines, where they showed partial dominance and expression dependent on the genetic background [10]. Furthermore, the expression of the *Ht* genes is modified by the environment, particularly temperature and light intensity [11]. In addition, qualitative resistances conferred by single genes such as the *Ht* genes tend to be overcome by new, virulent races of *Setosphaeria turcicae.g.*[12,13]. All these aspects limit the practical value of the *Ht* genes and have hampered their use in maize breeding programs.

Quantitative resistances are considered to be oligo- or polygenically inherited and, thus, partially as well as moderatly effective, but race unspecific and durable (for review see [14]). Due to the latter two properties, quantitative resistances are today considered more useful in a breeding context than qualitative resistances. In agrement with this conclusion, the majority of disease resistances deployed in elite varieties of maize are quantitative. However, identification of genes confering quantitative resistance is much more challenging than identifying R genes, owing to their smaller phenotypic effects.

Various studies have been conducted to map quantitative trait loci (QTLs) for resistance to NCLB (for review see [15]). All of them were linkage mapping studies using different types of progenies such as *F*_2_ or *F*_3_ generations, *BC*_1_ generations, or populations of near isogenic lines or recombinant inbred lines. In these studies, QTLs were detected on all maize chromosomes except chromosome ten. Due to the large confidence intervals of QTLs and a restricted allelic sampling in the two parental genotypes, however, the results of linkage mapping studies had so far little impact on resistance breeding. Very recently, NCLB resistance in maize was dissected using the nested asociation mapping (NAM) population [16], which offers the advantage of a higher mapping resolution and a broader allelic sampling than the above mentioned linkage mapping studies. Nevertheless, population-based association mapping has the potential of resulting in an even higher mapping resolution and broader allelic sampling compared to NAM [17]. To our knowledge, however, no genome-wide population-based association mapping study has been yet conducted for NCLB resistance in maize.

Resistance genes identified by linkage or association mapping might affect the disease either directly or indirectly (*cf.*[18,19]). Genes affecting plant growth and development or time to flowering (FT) fall in the latter class. Especially for diseases caused by necrotrophic pathogens such as *Setosphaeria turcica*, which are more severe on senescing leaf tissue after anthesis, a relationship betweeen plant disease resistance and FT might be expected [20]. Despite the contradictory results from earlier phenotypic analyses (*e.g.*[21,22]), some QTLs for NCLB resistance found in meta-analyses colocalized with those for FT and maturity (for review see [15]). However, in contrast to these linkage mapping studies, our association analysis will allow to discriminate with a high mapping resolution between pleiotropy and linkage of QTL for NCLB resistance and FT (*cf.*[23]).

In this study, a large association mapping panel comprising 1487 elite maize inbred lines was used to (i) identify chromosomal regions affecting FT and NCLB resistance, (ii) examine the epistatic interactions of the identified chromosomal regions with the genetic background on an individual molecular marker basis, and (iii) dissect the correlation between NCLB resistance and FT.

## Results

**Figure 1:**
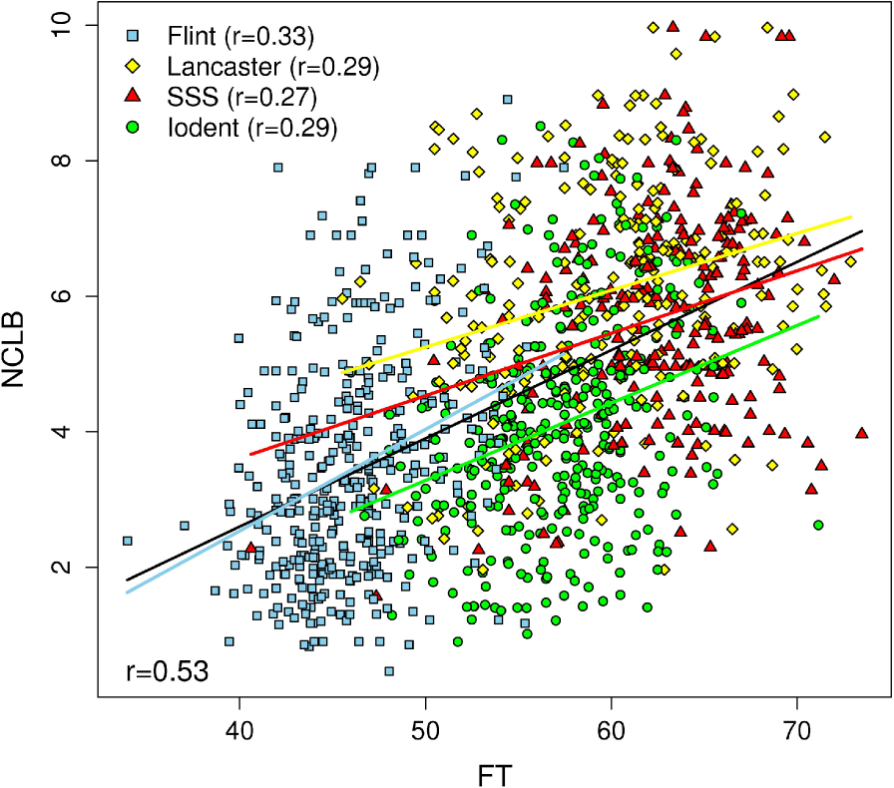
Regression curves of the adjusted entry means of flowering time (FT) vs. northern corn leaf blight resistance (NCLB) for the entire set of 1487 maize inbred lines as well as the individual heterotic pools. r is Pearson’s correlation coefficient between the two traits.

For the whole set of phenotyped and genotyped inbred lines, the heritability of FT and NCLB resistance was 0.95 and 0.85, respectively (Table 1). NCLB was significantly (*r *= 0.53, *α *= 0.05) correlated with FT (Figure 1) in the whole s et of 1487 inbred lines. The Pearson correlation coefficient was lower within the four heterotic pools and ranged from 0.27 (Stiff Stalk; SSS) to 0.33 (Flint) (Figure 1).

**Table 1:** First and second-degree statistics for flowering time (FT) and northern corn leaf blight (NCLB) resistance of the 1487 phenotyped and genotyped inbred lines

	**Trait**	
**Parameter**	**FT**	**NCLB**
mean(*M*_*i*_)	55.2	4.8
range(*M*_*i*_)	34.0−73.5	0.5−10.0
$\sigma_{G}^{2}$	42.34	3.24
$\sigma_{G.E}^{2}$	2.21	0.63
*h*^2^	0.95	0.87

FT is measured in number of days to female flowering after June 1.

NCLB is rated from 1-9 (sensitive-resistant).

*M*_*i *_is the adjusted entry mean of genotype *i* calculated across all environments.

$\sigma_{G}^{2}$ and $\sigma_{G.E}^{2}$ are the genotypic and genotype × environment interaction variances.

*h*^2^ is the heritability on entry an mean basis.

**Figure 2:**
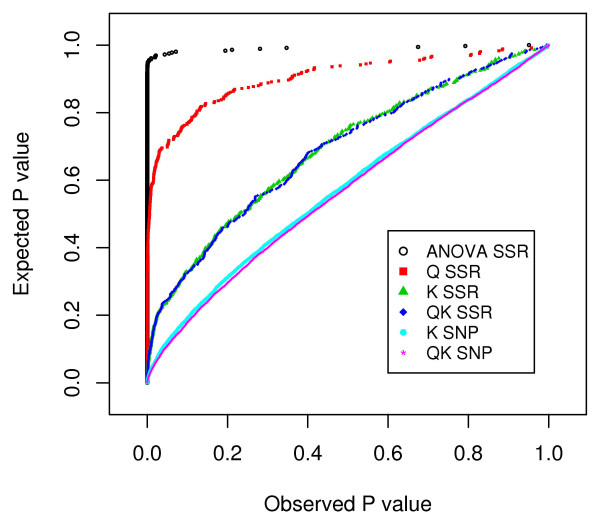
**Observed vs. expected*P*values for different two-step association mapping methods of northern corn leaf blight resistance with simple sequence repeat (SSR) and single nucleotide polymorphism (SNP) markers.**

For the SSR markers, the observed *P* values obtained with the QK and K model showed in comparison to the ANOVA and the Q model a smaller deviation from the uniform distribution (Figure 2). Furthermore, the mean squared difference (MSD) between observed and expected *P* values was slightly smaller for the QK model than for the K model. In addition to the SSRs, this was also true for the SNP markers (0.041 versus 0.042; 0.005 versus 0.007, respectively). The population background structure accounted for 21, 6, and 2% of the genetic variation in FT, NCLB and NCLB resistance corrected for FT (NCLB_*FT*_), respectively.

**Figure 3:**
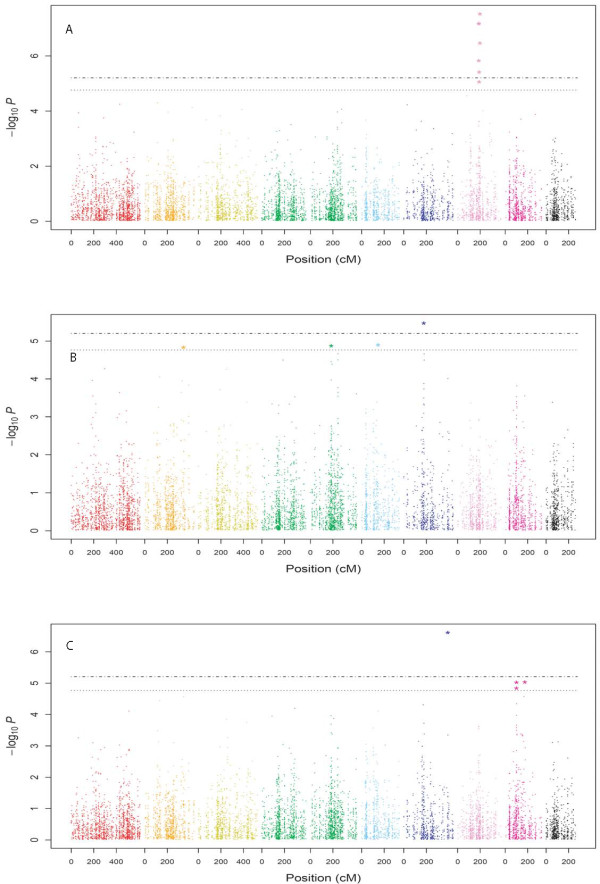
**Genome-wide *P* values for association analysis of flowering time (FT; A), northern corn leaf blight (NCLB; B), and FT corrected NCLB resistance (C) for the entire set of 1487 maize inbred lines.** The ten colors represent the ten chromosomes. The horizontal, doted and dashed-doted lines correspond to a nominal 5% significance threshold with Bonferroni and amplicon-wise Bonferroni correction, respectively. Significant *P* values are represented by a star.

In single marker analyses, seven, four, and four SNP markers were significantly (*α *= 0.05, amplicon wise Bonferroni correction) associated with FT, NCLB, and NCLB_*FT*_resistance, respectively (Figure 3). For FT, the seven SNPs explained individually between 5.39 and 14.29% of the genetic variance, whereas all SNPs together explained 13.20% (Table 2). For NCLB and NCLB_*FT*_, the four SNPs explained between 3.32 to 4.78% and between 0.36 to 6.76% of the genetic variance, respectively. In a simultaneous fit, they explained 8.18 and 9.48% of the genetic variance of NCLB and NCLB_*FT*_, respectively.

**Table 2:** Single nucleotide polymorphism (SNP) marker loci significantly associated with flowering time (FT), northern corn leaf blight (NCLB), and FT corrected NCLB (NCLB_*FT*_) resistance for the entire set of 1487 maize inbred lines as identified by single marker analysis

	**Marker**		**Chr.**	**Position**	**Position**		**Allele**	**Effect**	***p***_***G***_
**Trait**	**locus**	**Gene**	**bin**	**(cM)**	**(bp)**	***P* value**	**1/2**	**Allele 1-2**	**(%)**
FT	M_00048149	LE00126	8.05	187.40	128429853	1.5e-06^**^	G/A	1.66	10.72
	M_00041827	AY104033	8.05	188.17	130730508	6.8e-08^**^	C/T	2.21	9.75
	M_00041828	AY104033	8.05	188.17	130730581	6.6e-08^**^	C/T	2.21	9.81
	M_00044984	CL4016	8.05	191.34	131678990	3.8e-06^**^	T/C	1.49	6.34
	M_00044985	CL4016	8.05	191.34	131678953	8.7e-06^*^	A/G	1.43	5.39
	M_00049486	LE00214	8.05	197.92	145084250	3.0e-08^**^	C/T	-2.03	14.29
	M_00049487	LE00214	8.05	197.92	145084209	3.4e-07^**^	C/A	-1.66	9.10
	Simultaneous fit								13.20
NCLB	M_00040077	AY105483	2.08	343.92	217079800	1.4e-05^*^	G/A	-0.54	3.32
	M_00041320	AY112216	5.03	185.02	67600206	1.3e-05^*^	C/T	-0.50	3.58
	M_00043741	AY111579	6.05	146.44	145332550	1.3e-05^*^	C/T	0.61	4.27
	M_00048400	LE00018	7.02	178.00	100239763	3.3e-06^**^	T/C	-0.47	4.78
	Simultaneous fit								8.18
NCLB_*FT*_	M_00045254	AY107778	7.04	391.46	169937062	2.4e-07^**^	T/C	-1.89	0.36
	M_00041355	AY107035	9.03	99.76	43679500	1.4e-05^*^	A/C	-0.58	6.76
	M_00041356	AY107035	9.03	99.76	43679530	9.3e-06^*^	G/A	-0.58	6.66
	M_00039528	AY104217	9.05	174.09	135147555	9.1e-06^*^	A/G	-0.72	4.04
	Simultaneous fit								9.48

p_G_ is the proportion of the explained genotypic variance.

^*^Significant at *P* < 0.05 with amplicon-wise Bonferroni correction.

^**^Significant at *P* < 0.05 with Bonferroni correction.

In the Flint, Lancaster, SSS, and Iodent pool, two, four, two, and six SNPs were significantly (*α *= 0.05, amplicon wise Bonferroni correction) associated with FT (Additional file 1: Figure S1), which explained in a simultaneous fit 1.87, 22.99, 21.35, and 25.50% of the genetic variance in the corresponding heterotic pools (Table 3). For NCLB, two and six significantly associated SNP markers were identified in the SSS and Iodent pool, respectively, but none for the Flint and Lancaster pools (Additional file 2: Figure S2). Similarly, one and three SNPs were found to be significantly associated with NCLB_*FT*_ in the SSS and Iodent pool, respectively (Additional file 3: Figure S3). The SNPs associated with NCLB explained in a simultaneous fit 9.38 and 28.94% of the genetic variance, whereas those associated with NCLB_*FT*_explained 0 and 23.20% of the genetic variance in the SSS and Iodent pool, respectively (Table 3).

**Table 3:** Single nucleotide polymorphism (SNP) marker loci significantly associated with flowering time (FT), northern corn leaf blight (NCLB), and FT corrected NCLB (NCLB_*FT*_) resistance in the different heterotic pools as identified by single marker analysis. *p*_*G *_is the proportion of the explained genotypic variance and SSS is the Stiff Stalk heterotic pool

		**Marker**		**Chr.**	**Position**	**Position**		**Allele**	**Effect**	***p***_***G***_
**Trait**	**Pool**	**locus**	**Gene**	**bin**	**(cM)**	**(bp)**	***P* value**	**1/2**	**Allele 1-2**	**(%)**
FT	Flint	M_00049486	LE00214	8.05	197.92	145084250	4.3e-06^**^	C/T	-2.73	1.52
		M_00047185	LE00173	9.02	75.54	18327094	5.2e-06^**^	A/T	-3.61	0.00
		Simultaneous fit								1.87
	Lancaster	M_00045062	MAGI39472	3.07	346.79	195315842	1.5e-05^*^	C/T	2.83	7.48
		M_00045063	MAGI39472	3.07	346.79	195315842	1.5e-05^*^	T/G	2.83	7.48
		M_00048149	LE00126	8.05	187.40	128429853	5.8e-06^**^	G/A	3.15	10.20
		M_00049487	LE00214	8.05	197.92	145084209	1.9e-06^**^	C/A	-4.20	13.64
		Simultaneous fit							22.99	
	SSS	M_00048750	LE00097	2.02	95.07	5820265	2.4e-06^**^	G/A	13.62	14.80
		M_00047756	LE00008	5.03	186.65	67510540	5.6e-06^**^	C/T	-6.25	16.76
		Simultaneous fit								21.35
	Iodent	M_00039634	AY106491	8.03	115.95	65244298	1.7e-05^*^	G/A	3.57	18.18
		M_00044984	CL4016	8.05	191.34	131678990	1.1e-05^*^	T/C	1.95	14.82
		M_00044985	CL4016	8.05	191.34	131678953	1.2e-05^*^	A/G	1.98	15.18
		M_00040388	AY106357	8.05	192.10	134066305	1.2e-06^**^	T/C	2.51	17.14
		M_00042146	AY109558	8.05	192.10	135061056	1.3e-05^*^	T/C	2.30	18.77
		M_00046254	HDT102	8.05	192.26	136131540	1.4e-05^*^	T/A	-2.16	17.55
		Simultaneous fit								25.50
NCLB	SSS	M_00046267	HAM101	2.08	322.28	212539314	1.2e-06^**^	C/T	1.09	9.38
		M_00046268	HAM101	2.08	322.28	212539211	7.3e-07^**^	T/C	1.10	11.30
		Simultaneous fit								9.38
	Iodent	M_00048759	LE00099	3.05	227.44	160670670	1.4e-05^*^	A/G	1.78	6.05
		M_00044733	AZM452718	5.03	187.81	69119810	5e-06^**^	G/A	-0.90	8.49
		M_00046712	AY103770	9.03	111.71	99416354	6e-06^**^	G/C	1.25	10.33
		M_00046713	AY103770	9.03	111.71	99416416	9.7e-07^**^	T/G	-1.32	11.49
		M_00040630	AY105678	9.05	151.55	130918789	9.1e-06^*^	C/T	-0.81	10.02
		M_00040631	AY105678	9.05	151.55	130918887	1.1e-05^*^	T/C	-0.79	9.08
		Simultaneous fit								28.94
NCLB_*FT*_	SSS	M_00046331	HAM101	2.08	322.10	212537417	5.9e-06^**^	A/T	-1.01	0.00
		Simultaneous fit								0.00
	Iodent	M_00040577	AY105760	9.03	99.89	50762538	1.3e-05^*^	T/G	0.90	12.87
		M_00040630	AY105678	9.05	151.55	130918789	5e-07^**^	C/T	-0.97	18.89
		M_00040631	AY105678	9.05	151.55	130918887	5.3e-07^**^	T/C	-0.96	15.99
		Simultaneous fit								23.20

^*^Significant at *P* <0.05 with amplicon-wise Bonferroni correction.

^**^Significant at *P* <0.05 with Bonferroni correction.

**Figure 4:**
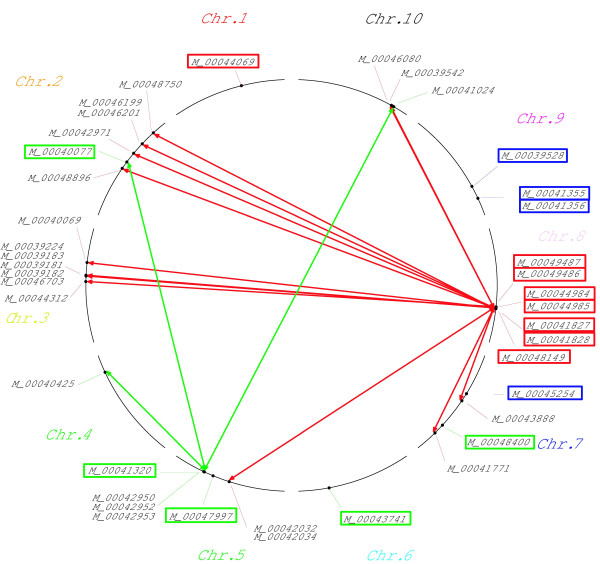
**Epistatic networks for flowering time (FT; red), northern corn leaf blight (NCLB; green), and FT corrected NCLB (blue).** The markers showing main effects in the single marker analysis and the multiple forward regression are framed with the respective colors.

The three rounds of multiple forward regression revealed for the whole set of 1487 inbred lines three SNP markers to be significantly associated with FT and NCLB, but only two with NCLB_*FT*_ (Table 4). The simultaneous fit of these SNPs explained 16.65, 7.62, and 6.13% of the genetic variance of FT, NCLB, and NCLB_*FT*_, respectively. Significant (*α *= 0.05, amplicon wise Bonferroni correction) epistatic interactions were identified between the significant SNPs from the single marker analyses as well as the multiple forward regression procedure and all other SNPs for FT and NCLB resistance, respectively (Figure 4). No significant epistatic interactions were detected for NCLB_*FT*_. The epistatic interactions found for the two traits explained a maximum of 5% of the genetic variance (Additional file 4: Figure S4).

**Table 4:** Simultaneous fit of single nucleotide polymorphism (SNP) markers identified by three rounds of multiple forward regression to be significantly (**α **= 0.05, amplicon wise Bonferroni correction) associated with flowering time (FT), northern corn leaf blight (NCLB), and FT corrected NCLB (NCLB_*FT*_) resistance for the entire set of 1487 maize inbred lines. p_G_ is the proportion of the explained genotypic variance

	**Marker**		**Chr.**	**Position**	**Position**	**Allele**	**Effect**	***p***_***G***_
**Trait**	**locus**	**Gene**	**bin**	**(cM)**	**(bp)**	**1/2**	**Allele 1-2**	**(%)**
FT	M_00049486	LE00214	8.05	197.92	145084250	C/T	-1.99	
	M_00041828	AY104033	8.05	188.17	130730581	C/T	1.93	
	M_00044069	MAGI31942	1.07	430.42	207180270	G/A	2.87	
								16.65
NCLB	M_00048400	LE00018	7.02	178.00	100239763	T/C	-0.49	
	M_00041320	AY112216	5.03	185.02	67600206	C/T	-0.51	
	M_00047997	LE00080	5.05	245.04	181521588	A/C	0.63	
								7.62
NCLB_*FT*_	M_00045254	AY107778	7.04	391.46	169937062	T/C	-1.91	
	M_00039528	AY104217	9.05	174.09	135147555	A/G	-0.73	
								6.13

## Discusssion

### Statistical aspects of association analysis

#### One-step vs. Two-step approaches

In all genetic mapping experiments, the one-step approach, in which phenotypic and genotypic data are analysed in a single step, is the only fully efficient analysis [24]. However, a comparison with the two-step analysis showed only a marginal increase in the empirical type I error rate [25]. As the two-step analysis is computationally much less demanding, we used this approach in view of the large data set analysed in our study.

#### Alternative association mapping models

Several methods for association analysis in plants have been described recently [25-27]. In order to identify the most appropriate association mapping method for our data set, we compared for background SSR markers several models with respect to the deviation of the *P* values from a uniform distribution [28]. This is because under the assumption that our SSR markers are unlinked to functional polymorphism due to their low genome coverage [29], it is expected that the *P* values observed for an association mapping approach are uniformly distributed (cf. [26]). The mean of squared difference (MSD) between observed and expected *P* values of all marker loci was therefore calculated as a measure for the deviation of the *P* values from a uniform distribution. The results of these analyses (Figure 2) suggested that the QK method [26] with kinship matrix **K** calculated as the fraction of shared alleles [27] was the most appropriate method for our data set with respect to the adherence to the nominal *α* level. The use of the QK method for the SNP-phenotype association analysis, however, resulted in fewer associations compared to the K method (data not shown). Because it is not possible to determine whether these associations were lost due to the lower power of the QK method or as they are caused by population structure, we decided for the conservative way and discussed below only the results of the QK method.

An alternative to single marker analysis is haplotype-based association analysis. This requires the building of haplotypes based on the extent of LD between the single markers. In the germplasm examined in our study, the average extent of LD between SNPs within amplicons varied from *r*^2^ = 0.253 to *r*^2^ = 0.304, depending on the heterotic pools investigated [29]. In the case of such relatively low levels of LD, the number of haplotypes per amplicon is high and therefore their frequencies low. This in turn leads to a low power for detecting associations by a haplotype-based analysis. Therefore, we think haplotype-based association mapping is no promising strategy in the case of our study.

#### Corrections for multiple testing

In genome-wide association mapping studies with *n* molecular markers, the same statistical test is performed *n* times at the significance level *α*. Across all tests, however, the experimental type I error rate will be much higher than *α* (*e.g.*[30]). To overcome this problem and obtain an appropriate significance threshold, it was recommended to apply the Bonferroni correction [31], where the *α *level is divided by the number of independent tests. However, determining the number of independent tests is not straight forward in the context of genome-wide association mapping studies. Owing to the correlation structure among markers, it would be overly conservative to use the total number of markers as a substitute for the number of independent tests [32]. As the 8 244 SNP markers of our study were derived from 2 973 amplicons and SNPs from the same amplicon tend to show higher correlations than SNPs from different amplicons [29], we used besides the total number of SNPs also the number of amplicons as correction factor for the Bonferroni procedure.

#### Single marker analysis vs. multiple forward regression

An efficient approach to identify significant marker-phenotype associations inspite of the collinearity between markers might be multiple forward regression (*cf.*[33]). We applied this approach in the context of mixed-model analyses and detected SNPs that have not been detected with the single marker analysis (Table 4). Furthermore, the three SNPs identified with the former method for FT explained a higher proportion of the genetic variance than those identified by using the latter method. These results corroborate the appropriateness of multiple forward regression procedures for association analyses.

For NCLB and NCLB_*FT*_, the SNPs identified by this approach, however, explained in a simultaneous fit a lower proportion of the genetic variance than the SNPs identified by the single marker analysis (Table 2; 4). This finding might be explained by the significance levels applied during the single marker analysis, which are not directly comparable to those of the multiple forward regression. Furthermore, since multiple forward regression for mixed-model approaches is computationally demanding, we were able to perform only three selection steps resulting in a maximum of three selected SNP markers and this provides another explanation for our findings. Therefore, in order to take full advantage of multiple forward regression, more efficient computation algorithms are required.

### Identified SNP-phenotype associations

In the entire germplasm set, the population structure explained 21% of the genetic variation of FT. This finding suggested that sufficient genetic variation remains for detection of SNP-FT associations. For FT, we observed for the single marker analysis a strong *P* value peak on bin 8.05, which comprised seven SNPs from four genes (Figure 3). Furthermore, this region was identifed by the multiple forward regression approach (Table 4). Earlier studies recognized this chromosomal region as a hot spot for FT QTLs and genes ( [34,35] and references cited in there). The physical map positions of the significantly associated SNPs ranged from 128 429 853 to 145 084 250 bp. The observed *P* value peak at about 130 Mbp is in proximity to *Vgt1*, a non-coding sequence regulating the flowering time gene *ZmRap2.7*[36]. However, the close consideration of that region revealed an additional *P* value peak at about 145 Mbp (Additional file 5: Figure S5). This observation might suggest that in addition to *Vgt1* a second gene could be involved in FT control in this region. However, the region identified in our study does not correspond to *Vgt2*[37], as the latter FT QTL has been mapped to the other side of *Vgt1* towards the top of the chromosome. Since the average linkage disequilibrium (LD) among the significantly associated SNPs in this region was high, these SNPs are not necessarily located in the causal genes, but the association might be due to SNPs in strong LD with polymorphisms in the causal genes (*cf.*[38]). This, however, requires further research.

Another gene that is frequently proposed to contribute to variation of FT in maize is *Dwarf8* (*D8*) (*e.g.*[39]). Even though our study included six SNPs from *D8*, we did not find any significant association in bin 1.10 where *D8* is located. Our observation is in accordance with the results of [40], who observed no significant association for *D8* in a set of European maize inbred lines. These findings might be explained by a correlation of the allele frequencies of polymorphisms in *D8* with population structure in the examined germplasm. When correcting for population structure, it will be impossible to identify such polymorphisms in association analyses [40].

In addition to the SNPs from the *Vgt1* region, we identified based on the multiple forward regression approach a SNP from bin 1.07 to be significantly associated with FT (Table 4). This SNP might be located in the QTL (near SSR umc1833) upstream of *D8* detected in a meta-analysis [34] and appears in a *P-type R2R3 Myb* transcription factor. Since various transcription factors such as *LHY *[41] or *CCA1*[42] are known to regulate FT in model species, our finding might suggest that this gene is functionally involved in FT regulation of maize.

In conclusion, we observed for FT in maize a very well interpretable pattern of SNP associations that is in harmony with previous genetic analyses. This illustrates that data from practical plant breeding programs can be used not only to dissect oligogenic [43] but also polygenic traits. Furthermore, our findings suggest that the SNP-NCLB associations described below might be successfully used in marker-assisted selection programs. We identified five genome regions (four from single marker analyses, one from multiple forward regression) to be significantly associated with NCLB resistance (Table 2; 4) which is considerably lower than the number of genome regions identified by [16]. This finding is most probably due to the different significance thresholds and study designs used.

None of the associations found in our studies was located in bin 8.05, where earlier studies mapped the qualitative NCLB resistance genes *Ht2* and *Htn1*[7,8]. Both these genes have been identified in exotic germplasm (Australia, Mexico) and, thus, the resistance alleles might be absent in European elite germplasm. Furthermore, converted inbred lines carrying these introgressed qualitative resistance genes were not included in our study in order to prevent any complications with the identification of quantitative resistance genes.

One SNP identified to be significantly associated with NCLB was located in bin 2.08 where the qualitative resistance genes *Ht1* and *HtP* have been identified [5,6] and where a QTL was found by [16]. The physical map positions of *Ht1* and this SNP, however, differ by about 10 Mbp. Nevertheless, the SNP is located within the interval made up by the two closest flanking markers of *HtP*[6]. Whether this gene, coding for a nonspecific lipid-transfer protein 3 precursor, contributes directly to NCLB resistance or is in LD with the causal gene warrants further research. The same was true for the SNP located in a gene of unknown function in bin 6.05, which resides within the confidence interval of a QTL affecting the incubation period (IP) of NCLB in maize [44] and was located close by a QTL affecting NCLB resistance and IP [16].

Three SNPs significantly associated with NCLB resistance were located in bins 5.03, 5.05, and 7.02 (Table 2; 4) and in each case, a distinct peak of *P* values was observed (Figure 3). Since all regions have been previously reported to contribute to variation in NCLB resistance [15,45,46], this finding suggests that the identified SNPs are either located in or closely linked with the causal genes (*cf.*[47]). In contrast to the SNP in bin 5.03, which is located in a gene of unknown function, the SNP in bin 5.05 is located in *GPC4*, a member of the *glyceraldehyde-3-phosphate dehydrogenase* gene family, which is involved in sugar metabolism and shows expression differences upon anaerobiosis as well as heat shock [48]. [16] found also a QTL in this region for which a candidate gene was an *aldehyde dehydrogenase*. The SNP in bin 7.02 is located in a *DBF1* like gene, which is a member of the *Apetala 2*/Ethylene transcription factor family [49] and supposed to have a function in abiotic stress responses and especially dessication tolerance [49,50].

### Dissecting the correlation between FT and NCLB

The results of our study indicated that FT and NCLB resistance are correlated across all heterotic pools (*r *= 0.53, Figure 1). This correlation can be explained by the fact that NCLB is a necrophytic disease and, thus, tends to progress more rapidly on senescing tissues [20]. However, the correlations in the individual heterotic pools were only moderate (Flint: 0.33, Lancaster: 0.29, SSS: 0.27, and SSS: 0.29). This suggests that the overall correlation relies to a substantial part on the differences between the heterotic pools with respect to FT and NCLB resistance trait values (Figure 1). Our observation explains why we found neither for the whole set of genotypes (as we accounted for population structure) nor in the individual heterotic pools any overlap between SNPs associated with FT and NCLB (Table 2,3,4; Figure 3), and thus, no evidence of a pleiotropic effect of FT on NCLB resistance at the SNP level which is in accordance with results of [16].

Furthermore, we found no collocation between the SNPs associated with NCLB and NCLB_*FT*_(Table 2; Figure 3) for the whole set of genotypes. This finding suggested that some of the SNP-NCLB associations outlined above for genes involved in heat and drought response might be due to an indirect link of these two traits with NCLB resistance as well as FT. Indeed, plants sensitive to drought stress have a tendency to show early senescense symptoms, which, in turn, leads to a higher sensitivity to necrotrophic pathogens such as *Setosphaeria turcica*[20].

Nevertheless, we identified SNPs in bins 7.04, 9.03, and 9.05 to be significantly associated with NCLB_*FT*_. The first SNP was located in *GID1L2*, a gibberellin receptor. Since gibberelin plays a role in basal disease resistance of various plant species [51,52], our finding might suggest that this gene is functionally involved in NCLB_*FT *_resistance of maize.

The other two SNPs in bin 9.03 and 9.05 also significantly associated with NCLB_*FT*_ were located in genes with unknown function and a Sodium-Hydrogene exchanger, respectively, for which no obvious link to NCLB_*FT*_ is apparent. Nevertheless, we observed for both associations distinct *P* value peaks supporting the hypothesis that these genes might be the causal genes or closely linked to them.

### Congruency of identified associations across heterotic pools

For FT, we found in three of the four heterotic pools significantly associated SNPs in one (Flint) or two (Lancaster and Iodent) of the genes that where identified in the whole set of genotypes in the *Vgt1* region (Table 2,3; Additional file 1: Figure S1). In contrast, in the SSS pool, no significant association was detected for these loci. This is in accordance with earlier studies, in which QTLs were not detected in all examined populations in the *Vgt1* region [38,53,54]. One reason could be that in the SSS pool no LD was present in the region between the causal gene and the examined polymorphisms. Another explanation might be that the early allele of *Vgt1* does not occur in the SSS pool, because it flowers later than the other pools.

SNPs significantly associated with NCLB and NCLB_*FT*_resistance were found in the SSS and Iodent pools, but not in the Flint and Lancaster ones (Table 3; Additional file 2: Figure S2 and Additional file 3: Figure S3). One explanation could be the difference in the extent of LD between the heterotic pools. The LD decays more rapidly in the Flint and Lancaster pools compared to the two other pools resulting in a lower genome coverage of 13 and 48% vs. 207 and 121%. Furthermore, the number of markers required to detect associations explaining a significant part of the phenotypic variation (17 000 and 65 000; respectively) in the Flint and Lancaster pools is higher than the number of SNPs actually available [29]. This could limit the power to detect associations for NCLB and NCLB_*FT *_resistance in these two pools, whereas the number of required markers for the SSS and Iodent pools (4 000 and 7 000; respectively) is predicted to be sufficient.

In addition to the above described reasons for the imperfect congruency of the identified associations across heterotic pools are on one side sampling effects [55] but on the other side also epistatic interactions. Therefore, we searched for epistatic interactions between the significant SNPs identified in the whole set of genotypes and all the other markers. For FT and NCLB, highly significant epistatic interactions were detected (Figure 4) suggesting that epistasis contributes to the imperfect congruency of identified associations across different heterotic pools. This was even more important for NCLB, for which the epistatic interactions between markers explained as much genetic variation as their main effects (Additional file 4: Figure S4). These results are contradictory to the results of [16], who didn’t find significant epistatic interactions between QTL markers and the others. The fact that elite breeding material was examined in our study, which has undergone a long process of selection, whereas the NAM population consists of multiple connected recombinant inbred line populations, could explain this difference.

### Relevance of the identified associations for practical breeding

The significant SNP-FT associations identified in our study explained about 15% of the genetic variance (Table 2). This value is much lower than the value reported by [35]. This difference is due to the fact that they used (i) a stepwise forward regression, (ii) segregating populations, and (iii) a total of 5 000 genotypes, which increase the power of QTL detection. In contrast to FT, the associations identified for NCLB resistance in our study explained only about 5% of the genetic variance (Table 2). This finding clearly suggests that the genetic architecture of NCLB has a higher genetic complexity than FT and, therefore, phenotypic but also marker-assisted selection will result in a lower gain of selection for the former than the latter. Nevertheless, for breeding applications, it seems more interesting to concentrate on NCLB_*FT *_rather than NCLB, because the former is corrected for FT, the detected SNPs explain even a higher proportion of the genetic variance compared to the latter, and the correlation with population structure is lower for the former than the latter.

The proportion of the explained genetic variance was generally much higher in the individual pools than in the entire germplasm set (Table 2, 3). Partly, this might be due to the reduced sample size leading to the overestimation of the allele effects and the explained genetic variance [56]. However, as the individual heterotic pools still comprise almost 400 genotypes, this overestimation is expected to be only small. More likely, our observation can be explained by different loci contributing to the variation of the examined traits in the individual heterotic pools (Table 3). Another explaination could also be the epistatic intereactions which importance differs among the heterotic pools. Finally, genome structure differences among the heterotic pools such as copy number or presence/absence variants [57] can explain our observation. Our finding suggests that despite association analysis across heterotic pools might be relevant for some traits to unravel the genetic architecture, marker-assisted selection within the individual heterotic pools, as praticed by plant breeders, is more promising than across heterotic pools.

Although we observed for FT highly significant epistatic interactions, these explained only a low proportion of the genetic variance compared to the main effects and, therefore, might be disregarded in marker-assisted selection for this trait. However, this was not true for NCLB as the epistatic interaction explained partly a higher proportion of the genetic variance than the main effects. Thus, taking epistasis into account for this trait should increase the efficiency of marker-assisted selection (Additional file 4: Figure S4).

## Conclusions

We observed for FT, a trait for which already various genetic analyses in maize have been performed, a very well interpretable pattern of SNP associations, suggesting that data from practical plant breeding programs can be used to dissect polygenic traits. Furthermore, we described SNPs associated with NCLB and NCLB_*FT *_resistance that are located in genes for which a direct link to the trait is discernable or which are located in bins of the maize genome for which previously QTLs have been reported. Some of the SNPs showed significant epistatic interactions with markers from the genetic background. The observation that the listed SNPs and their epistatic interactions explained in the entire germplasm set about 10% and in the individual heterotic pools up to 30% of the genetic variance suggest that significant progress towards improving the resistance of maize against NCLB by marker-assisted selection is possible with these markers, without much compromising by a late flowering time. Furthermore, these regions are interesting for further research to understand the mechanisms of resistance to NCLB and diseases in general, because some of the genes identified were not annotated so far for these functions. However, as association mapping provides only statistical, *i.e.*, indirect evidence for the function of the identified gene [58], a direct proof of the function of the identified alleles is still necessary.

## Methods

### Plant materials, field experiments

Our investigation was based on a set of 4 149 maize inbred lines representing elite European and North American germplasm. The inbred lines are proprietary to the plant breeding company Limagrain (France) and were assigned by breeders to four heterotic pools, namely Flint, Lancaster, SSS, and Iodent.

In the years 2000 to 2009, these genotypes were evaluated for their *per se* performance in routine plant breeding trials, at different numbers of locations (2-7), with different experimental designs (randomised complete block design, nested design, *etc.*) and numbers of replicates (1-3). The experiments were either naturally infested or artificially infested with *Setosphaeria turcica* according to standard protocols [59]. All entries were evaluated for FT and NCLB resistance. FT was recorded in number of days to silking after June 1. NCLB was rated on a scale from 1 (sensitive) to 9 (resistant) at the level of individual plots.

### Molecular marker assays

A subset of 1 487 inbred lines randomly selected from the phenotyped inbreds regarding FT and NCLB were analyzed with 359 SSR and 8 244 SNP markers (for details see [60]). The SSRs were selected over years by Limagrain with respect to their polymorphism information content value [61] in various sets of maize inbreds. The SNPs were discovered by sequencing 2 973 amplicons in a development set of 30 diverse maize inbreds. From these, SNPs which showed an Illumina designability score > 0.4 and were not in complete LD in the development set, were selected for genotyping the 1 487 lines. The proportion of missing data was 5.1% for the SSRs and 2.7% for the SNPs. The amplicons had an average size of 477 bp and contained on average three SNPs.

All markers were mapped in the IBM population [62]. Chromosomes 1 to 10 carried 59, 42, 41, 34, 36, 31, 36, 31, 27, and 22 of the SSR markers, respectively. In addition, 1 456, 858, 902, 898, 1 002, 633, 578, 632, 699, and 586 of the SNPs were mapped to chromosomes 1 to 10, respectively. The total map length was 4 265 cM for the SSRs and 4 378 cM for the SNPs. The physical positions of the markers were extract from *Zea mays* Genome Browser - Release 2.0.

Genotyping of the SSRs was performed by Limagrain Verneuil Holding (Riom, France) using standard protocols. Genotyping of the SNPs was performed by Biogemma (Clermont-Ferrand, France) using an Illumina Infinium iSelect chip.

### Statistical analyses

#### Phenotypic data analyses

Phenotypic data were analysed based on the following mixed model:

(1) $$\begin{array}{l} y_{\text{ijklm}}\;=\;\mu +g_{i}+u_{j}+g_{i}*u_{j}+\theta_{j}t_{\text{jk}}\\ \;\;+\beta_{\text{jk}}b_{\text{jkl}}+\rho_{\text{jkl}}r_{\text{jklm}}+e_{\text{jklm}}, \end{array}$$

where *y*_*ijklm*_ is the phenotypic observation for the *i*^*th*^ maize inbred line at the *j*^*th*^ environment (year-location combination) in the *m*^*th *^replicate of the *l*^*th *^block in the *k*^*th*^ trial, *μ* the intercept, *g*_*i*_ the genetic effect of the *i*^*th*^ maize inbred line, *u*_*j*_ the effect of the *j*^*th*^ environment, *g*_*i *_∗*u*_*j*_ the genotype-by-environment interaction, *t*_*jk*_ the effect of the *k*^*th *^trial in the *j*^*th *^environment, *b*_*jkl*_ the effect of the *l*^*th *^block in the *k*^*th *^trial of the *j*^*th *^environment, *r*_*jklm*_ the effect of the *m*^*th *^replicate of the *l*^*th *^block in the *k*^*th *^trial of the *j*^*th *^environment, and *e*_*jklm *_the residual. *θ*_*j *_was a dummy covariate of value 1 in environments with several trials and of value 0 alternatively, *β*_*jk*_ a dummy covariate of value 1 in environments with several trials and blocks and of value 0 alternatively, and *ρ*_*jkl*_ a dummy covariate of value 1 in environments with several trials, blocks, and replicates and of value 0 alternatively.

Our study was based on data from 10 years and 23 locations spread over Europe, resulting in a total of 45 environments and, thus, the environmental factor was regarded as random. Error variances were assumed to be heterogeneous among environments. For calculating the adjusted entry mean *M*_*i*_ for each of the 4 149 inbred lines across all trials, we regarded *g*_*i *_as fixed and all other effects as random.

For estimation of variance components, except *μ*, all effects including *g*_*i *_were regarded as random. Heritability on an entry mean basis was calculated for the phenotyped and genotyped inbred lines according to [63] for unbalanced breeding trials.

NCLB_*FT*_was calculated according to [64]. A regression curve of NCLB against FT was computed (Figure 3). The vertical distance of an inbred’s adjusted entry mean to the regression curve represented its NCLB_*FT *_resistance value. Negative values indicated susceptible plants and positive values resistant plants.

Variance components were determined by the REML method. The mixed model analyses were performed with ASREML release 2.0 [65]. All other analyses were performed using the software R [66].

#### Association analyses

*Single marker analysis:* In the second step of our approach, we used the adjusted entry means for FT, NCLB, and NCLB_*FT*_ to test their associations with each of the 8 244 SNP markers, using the QK method [67]:

(2) $$M_{\text{ip}}=\mu +m_{p}+g_{i}^{*}+\overset{z}{\underset{u=1}{\sum }}D_{\text{iu}}v_{u}+\varepsilon_{\text{ip}},$$

where *M*_*ip*_ is the adjusted entry mean of inbred *i* carrying the *p*^*th *^allele, *m*_*p*_ is the effect of the *p*^*th*^ allele of the SNP marker under consideration, $g_{i}^{*}$ the residual genetic effect of the *i*^*th *^entry, *v*_*u*_ the effect of the *u*^*th*^ column of the population structure matrix **D**, and *ε*_*ip *_the residual. The variance-covariance matrix of the vector of random effects $g^{*}=\{g_{1}^{*},\ldots ,g_{1487}^{*}\}$ was assumed to be $\text{Var}(g^{*})=2\text{K}\sigma_{g^{*}}^{2}$, where **K**was a 1487×1487 matrix of kinship coefficients that define the degree of genetic covariance between all pairs of inbreds, and $\sigma_{g^{*}}^{2}$ is the genetic variance estimated by REML. The variance-covariance matrix of the vector of errors *ε*_*ip*_ was assumed to be $\text{Var}(\varepsilon )=\text{I}\sigma_{\varepsilon }^{2}$.

The population structure matrix **Q** was calculated based on SSR markers using the software STRUCTURE [68] as described in detail by [60]. Per definition, the *z* + 1 columns of the **Q** matrix add up to one. Thus, only the first *z* columns were used as **D**matrix in our study, to achieve linear independence and, thus, avoid singularities. The kinship matrix **K** was calculated as described by [27] based on the SSR markers. In addition to the above described QK approach, we also examined other models: ANOVA, Q, K and K_T_ (Additional file 6: Figure S6) for SNP markers but also for SSR markers [28]. In order to compare these different association mapping methods, expected *P* values were calculated and the MSD between observed and expected *P* values of all marker loci was then calculated as a measure of the deviation of the observed *P* values from the uniform distribution [25].

Based on the Wald statistics, we performed a test for the presence of significant (*α *= 0.05) SNP effects for each of the three traits. We dealt with the multiple testing problem by applying a Bonferroni and amplicon number based Bonferroni correction [31]. For the former, we used the total number of SNP markers to calculate the Bonferroni correction, whereas, for the latter, the correction was calculated using the number of amplicons from which the examined SNPs were derived. The proportion of the genetic variance explained by the significant SNPs was computed based on the relative reduction in genetic variance when the SNPs were added to the model [69]. Similarly, the proportion of genetic variance explained by the **D** matrix was calculated. Negative values were set to zero.

*Heterotic pools:* Similarly to the analyses conducted for the whole set of inbred lines, single marker analyses were conducted for each of the four heterotic pools. The same model was applied, except that no **D** matrix was considered in this case, as the population structure within the heterotic pools was modelled by the kinship matrix **K**.

*Multiple forward regression:* In order to take into account the LD between SNPs, we used in addition to the single marker analysis a multiple forward regression approach to identify, based on the above described QK model, those marker combinations which explain best the genotypic variation. A P-to-enter criterion was used. We added the SNP with the lowest *P* value in the single marker analysis (if significant according to the amplicon based Bonferroni correction), as fixed cofactor in the analyses, when examining all remaining SNP markers for their association with the phenotype. For each of the three traits, this prodedure was repeated due to the high computational burden only two times and, thus a maximum of three SNPs could be selected.

*Detection of epistasis:* For each of the three traits, we performed a screen for epistatic interactions between the significant SNPs from the single marker analysis as well as multiple forward regression and all other SNP markers. The multiple testing problem was considered using the two different Bonferroni corrections.

The association analyses of SSR markers were performed with ASREML release 2.0 [65], whereas the association analyses of SNPs were performed with EMMA [70].

## Author’s contributions

BS conceived the study. DVI performed all analyses and drafted the manuscript. BS, JPM, and AEM revised the manuscript. All authors read and approved the final version of the manuscript.

## Supplementary Material

Additional file 1 **Figure S1. Genome-wide *P* values for association analysis of flowering time within the different hererotic pools (Flint, Lancaster, SSS, Iodent, respectively).** The ten colors represent the ten chromosomes. The horizontal, doted and dashed-doted lines correspond to a nominal 5% significance threshold with Bonferroni and amplicon-wise Bonferroni correction, respectively. Significant *P* values are represented by a star. Click here for file

Additional file 2 **Figure S2. Genome-wide *P* values for association analysis of northern corn leaf blight resistance within the different hererotic pools (Flint, Lancaster, SSS, Iodent, respectively).** The ten colors represent the ten chromosomes. The horizontal, doted and dashed-doted lines correspond to a nominal 5% significance threshold with Bonferroni and amplicon-wise Bonferroni correction, respectively. Significant *P* values are represented by a star. Click here for file

Additional file 3 **Figure S3. Genome-wide *P* values for association analysis of flowering time corrected northern corn leaf blight resistance within the different hererotic pools (Flint, Lancaster, SSS, Iodent, respectively).** The ten colors represent the ten chromosomes. The horizontal, doted and dashed-doted lines correspond to a nominal 5% significance threshold with Bonferroni and amplicon-wise Bonferroni correction, respectively. Significant *P* values are represented by a star. Click here for file

Additional file 4 **Figure S4. Significant epistatic interactions between the most significantly associated SNP for flowering time (FT) and northern corn leaf blight (NCLB) resistance and all other SNP markers for the entire set of 1487 maize inbred lines. ***p*_*G*_ is the proportion of the explained genotypic variance. Click here for file

Additional file 5 **Figure S5. *P* values for association analysis of flowering time for the entire set of 1487 maize inbred lines on chromosome 8 in the *Vgt1* region.** Significant *P* values are represented by a star. Click here for file

Additional file 6 **Figure S6. Deviance of the QK mixed model association mapping method applied to northern corn leaf blight resistance of the entire germplasm set of the 1487 genotypes depending on threshold T.** For details, see Materials and Methods. Click here for file
